# Functional–structural relationship in large‐scale brain networks of patients with end stage renal disease after kidney transplantation: A longitudinal study

**DOI:** 10.1002/hbm.24804

**Published:** 2019-10-01

**Authors:** Hui J. Chen, Yun F. Wang, Jiqiu Wen, Qiang Xu, Guang M. Lu, Long J. Zhang

**Affiliations:** ^1^ Department of Medical Imaging Jinling Hospital, Medical School of Nanjing University Nanjing Jiangsu China; ^2^ Department of Radiology Affiliated Hainan Hospital of Hainan Medical College, Hainan General Hospital Haikou Hainan P.R. China; ^3^ National Clinical Research Center of Kidney Disease Jinling Hospital, Medical School of Nanjing University Nanjing Jiangsu China

**Keywords:** diffusion tensor imaging, end‐stage renal disease, graph theory analysis, kidney transplantation, large‐scale complex networks, resting‐state functional MRI

## Abstract

It is unclear how the brain network changed after kidney transplantation (KT). We explored the patterns of large‐scale complex network after KT in end‐stage renal disease (ESRD) patients with resting‐state functional MRI (rs‐fMRI) and diffusion tensor imaging (DTI). Twenty‐one ESRD patients (14 men; mean age, 31.5 ± 9.9 years) scheduled for KT and 17 age‐ and gender‐matched healthy controls (HC) (8 men; mean age, 28.9 ± 7.2 years) were enrolled in this study. Each participant underwent rs‐fMRI and DTI scans in three time points (pre‐KT, 1 and 6 months after KT [for ESRD]). Graph theory analysis was used to characterize the topological properties by using functional and structural network connectivities intergroup correlation analysis was performed between functional/structural MR indexes and clinical markers. Compared with HC, pre‐KT ESRD patients showed an altered topological organization in both functional and structural networks. Compared with pre‐KT, increased node degree and node efficiency were observed for both functional and structural networks at 1 month after KT (all *p* < .05), which were further increased at 6 months after KT (*p* < .05). Both functional and structural networks did not recover completely at 6 months after KT (all *p* < .05). The patients showed an increased functional–structural connectivity coupling at 1 month after KT compared with HC (*p* = .041). A trend of progressive recovery of functional and structural connectivity networks was observed in ERSD patients after KT, which did not recover to the normal levels even in 6 months after KT. The study results underlie cognitive function recovery in ESRD patients following KT in the neuropathophysiological perspective.

**Abbreviations**
DMN: default mode network
DST: digit symbol test
DTI: diffusion tensor imaging
ESRD: end stage renal disease
HC: healthy control
KT: kidney transplantation
LTT: line‐tracing test
NCT‐A: number connecting test type A
rs‐fMRI: resting‐state functional MR imaging
SAS: self‐rating anxiety scale
SDS: self‐rating depression scale
SDT: serial dotting test

## 1 INTRODUCTION

The human brain is a large‐scale complex network exhibiting “small‐world” properties, which are characterized by high clustering of a lattice and the short path length (Watts & Strogatz, 1998). These properties allow efficient dynamic interactions between spatially distinct areas and they are responsible for the high‐level information processing of the human brain. Graph theory analysis (He & Evans, 2010) offers a method to assess structural and functional human brain organization in vivo. With the ability to reveal the structural and functional connectivity of the large‐scale brain networks noninvasively, magnetic resonance imaging (MRI) offers the opportunity to better understand the pathophysiological, behavioral, and cognitive consequences of focal and diffuse brain diseases. With graph theory analysis, researchers found that the network properties change as the age increases (Achard & Bullmore, 2007; Fair et al., 2009). Brain structural and functional network topology has also been found to be abnormal in many neurological diseases, such as Alzheimer's disease (Supekar, Menon, Rubin, Musen, & Greicius, 2008), multiple sclerosis (Shu et al., 2011), temporal lobe epilepsy (Liao et al., 2010), and end stage renal disease (ESRD) (Ma et al., 2015). Structural and functional connectivities are reciprocally interdependent (Rubinov, Sporns, van Leeuwen, & Breakspear, 2009). Structural connectivity networks can provide details regarding structural architectural features and it is based on white matter tracts quantified by diffusion tractography or correlations of morphological measures. Functional connectivity networks calculate the temporal correlations or coherences between blood oxygen level‐dependent functional MRI (fMRI) signals from distinct brain regions and it offers a network perspective on brain dynamics. The structural connectivity network is always considered to be the physical substrate of the functional connectivity network. Functional connectivity network is considered to be more flexible. Simultaneous measures of the functional and structural connectivity networks and their relationship allow for better understanding of the alternations in brain connectivity (Sporns, 2011).

Cognitive impairment is prevalent in patients with ESRD (Seliger & Weiner, 2013). This is particularly true among those receiving hemodialysis. Cognitive impairment may lead to a poor quality of life and a remarkable increase in mortality (Seliger & Weiner, 2013). Previous studies have found that cognitive impairment in ESRD patients may be attributed to the changes of brain network (Ni et al., 2014). Ni et al. (2014) found that functional connectivity in the default mode network (DMN) was impaired and may be related to the reduced performance of neurocognitive tests in ESRD patients. Kong et al. (2014) also found that the reduced white matter integrity may be correlated with cognitive dysfunction in ESRD patients. All these studies indicate that brain functional and structural connectivity abnormalities had occurred in ESRD patients.

Kidney transplantation (KT) has been considered to be the most effective method in patients with ESRD for cognition improvement and different function domains recover at different periods (Harciarek, Biedunkiewicz, Lichodziejewska‐Niemierko, Debska‐Slizien, & Rutkowski, 2011). Nonetheless, the mechanism underling the cognition improvement has not yet been well elucidated. To the best of our knowledge, only three reports were published in this field (Chen et al., 2018; Gupta et al., 2016; Zhang et al., 2016). Zhang et al. (2016) found that functional connectivity changes in the DMN may recover earlier than structural connectivity changes 1 month after KT (KT‐1m). Gupta et al. (Gupta et al., 2016) found that improved cognitive function 3 months after KT was associated with white matter integrity improvements. Chen et al. (2018) found that resting‐state subnetworks exhibited variable recovery patterns, particularly DMN and the sensorimotor network did not recover completely 6 months after KT (KT‐6m). Nevertheless, the neuropathological substrate of cognitive improvement after KT in ESRD patients is far from well‐established, particularly at the whole‐brain network perspective.

The purpose of this study is thus to investigate dynamical topological reorganization of intrinsic whole‐brain functional and structural networks in ESRD patients after KT.

## 2 MATERIALS AND METHODS

### 2.1 Subjects

This prospective study was approved by the Medical Research Ethics Committee of our hospital, and the written informed consent of each subject was obtained before this study. From June 2012 to July 2015, 21 patients (17 patients underwent hemodialysis, 2 patients received peritoneal dialysis, and 2 patients did not receive any replacement therapy during baseline examinations) with ESRD (estimated glomerular filtration rate, <15 ml per minute per 1.73 m^2^ or currently receiving regular dialysis treatment) who were scheduled for KT were enrolled from National Clinical Research Center of Kidney Disease, Jinling Hospital, Medical School of Nanjing University. The average dialysis time was 18.9 ± 25.0 months (0–100 months). The hemodialysis patients received hemodialysis three times a week, while the peritoneal dialysis patients received peritoneal dialysis four times per day. All 21 patients completed all necessary laboratory examinations, neuropsychologic tests and MRI examinations before, 1 month and 6 months after KT. The following further exclusion criteria were applied: (a) history of any drug or alcohol abuse; (b) noticeable brain lesions such as stroke and tumor; (c) history of or current psychiatric disorders; (d) other systemic diseases; (e) clinically relevant visual or hearing difficulties; (f) previous KT or other organ transplantation; (g) drop‐outs; (h) unavailability for follow‐up at 1 month and 6 months after KT; and (i) head motion of more than 1.0 mm or 1.0° during MRI. None of the 21 patients was excluded according the above criteria.

Twenty‐three age and gender matched healthy control (HC) subjects were recruited as controls from the local community. They also completed all necessary laboratory examinations, neuropsychologic tests and MRI examinations at baseline, 1 month and 6 months follow‐up. Six HC subjects were excluded because of marked artifacts in the diffusion tensor imaging (DTI) data. Finally, 17 HC subjects were eligible. All 17 HC subjects had no systemic diseases and no history of psychiatric or neurologic diseases. They were self‐identified as right‐handed, and had normal eyesight.

### 2.2 Neuropsychological tests

All patients and HC underwent a battery of neuropsychological tests including the number connecting test type A (NCT‐A), digit symbol test (DST), line‐tracing test (LTT), and serial dotting test (SDT). These examinations were performed at 24 hr after dialysis. These neuropsychological tests are associated with the domains of psychomotor speed, attention, and visual memory (Bajaj, Wade, & Sanyal, 2009). Depression and anxiety were measured by using the Hospital Anxiety and Depression Scale (i.e., self‐rating anxiety and depression scales) (SAS and SDS) (Faravelli, Albanesi, & Poli, 1986; Zung, 1971). All these tests were performed within 1 hr before each MRI examination. Less time in NCT‐A, line‐tracing test, serial dotting test and lower scores on SAS and SDS reflect better performance, while higher DST scores indicate better performance.

### 2.3 Laboratory tests

All ESRD patients and HC completed laboratory tests to determine renal function, serum creatinine, urea levels, hematocrit, and hemoglobin within 24 hr before each MRI examination (before, 1 month and 6 months after KT).

### 2.4 MRI data acquisition

MRI data were acquired by using a 3‐T MRI scanner (TIM Trio; Siemens Medical Solutions, Erlangen, Germany) for all subjects. All subjects were told to be still, keep eyes closed, but stay awake during MRI examination. Foam padding was used to reduce head motion. T1‐weighted and T2 fluid‐attenuated inversion‐recovery anatomic datasets were obtained from each subject for the detection of silent brain lesions using the following two sequences: (a) axial T1 fast low‐angle shot sequence with 30 axial slices; slice thickness, 4 mm; slice gap, 0.4 mm; image matrix, 320 × 256; field of view (FOV), 240 × 240 mm^2^; repetition time (TR)/echo time (TE), 350 ms/2.46 ms and (b) axial T2 fluid‐attenuated inversion‐recovery sequence with 25 axial slices; slice thickness, 4 mm; slice gap, 1.2 mm; image matrix, 232 × 256; FOV, 220 × 220 mm^2^; TR/TE, 9,000 ms/93 ms; flip angle, 130°; inversion time, 2,500 ms, bandwidth 287 Hz/Px. Subsequently, high‐resolution three dimensional T1‐weighted anatomical images were acquired in the sagittal orientation using a magnetization‐prepared rapid gradient‐echo sequence on each subject: TR/TE, 2,300 ms/2.98 ms; flip angle, 9°; number of sections, 191; FOV, 256 × 256 mm^2^; acquisition matrix, 256 × 256; and section thickness, 1 mm. Functional data were obtained using a gradient‐echo echo‐planar sequence: slice thickness, 4 mm; slice gap, 0.4 mm; matrix, 64 × 64; FOV, 240 × 240 mm^2^; TR/TE, 2,000 ms/30 ms; flip angle, 90°. Each fMRI sequence contained 250 volumes, and each volume included 30 axial sections placed approximately along the anterior–posterior commissure line. Each fMRI scan lasted 500 s. The DTI images were obtained using a spin echo‐based echo planar imaging sequence in contiguous axial planes, including 20 volumes with diffusion gradients applied along 20 noncollinear directions (*b* = 1,000 s/mm^2^) and 1 volume without diffusion weighting (*b* = 0 s/mm^2^). Each volume consisted of 30 contiguous axial slices covering the whole brain (TR/TE = 4,100 ms/93 ms, FOV = 240 × 240 mm^2^, matrix size = 128 × 128, voxel size = 1.8 × 1.8 × 4 mm^3^). All MRI acquisitions were performed by one author (H.J.C. with 5 years of experience in resting‐state fMRI [rs‐fMRI]).

### 2.5 Brain network construction

All MRI data were analyzed by two authors (Q.X. and H.J.C. with 9 and 5 years of experience in rs‐fMRI). The functional and structural connectivity networks were conducted as described in previous studies (Liao et al., 2011; Zhang et al., 2011) (see Supplementary Materials). For DTI, whole‐brain fiber tracking was performed in native diffusion space for each subject using the interpolated streamline algorithm embedded in the Diffusion Toolkit (Mori, Crain, Chacko, & van Zijl, 1999).

### 2.6 Network analysis

Graph theory analyses were performed on functional and structural connectivity networks of the patients and HCs using the Gretna (http://www.nitrc.org/projects/gretna/) (Wang et al., 2015).

### 2.7 Small‐world properties and regional nodal characteristics

Small‐world parameters were originally described by Watts and Strogatz (1998). In this study, we explored the small‐world properties of weighted functional and structural connectivity networks (Achard & Bullmore, 2007; Lo et al., 2010; Wen et al., 2011). The path length between nodes *i* and *j* was described as the sum of the edge lengths along the path, where each edge's length was obtained by calculating the reciprocal of the edge weight, 1wij. The shortest path length *L*_*ij*_ between nodes *i* and *j* was defined as the length of the path with the shortest length between the two nodes. The weight characteristic shortest path length $L_{\mathrm{net}}^{w}$ of a network was quantified by a “harmonic mean” length between pairs, to overcome the problem of possibly disconnected network components. Formally, $L_{\mathrm{net}}^{w}$ is the reciprocal of the average of the reciprocals: $L_{\mathrm{net}}^{w}\;$= 11NN−1∑i=1N∑j≠iN1Lij where *N* is the number of nodes. The weight characteristic shortest path length quantifies the ability for information propagation in parallel.

Three measures of regional node centrality, including nodal degree ($S_{i}^{w}$), efficiency ($E_{i}^{w}$) and betweenness centrality ($b_{i}^{w}$) were assessed as described in one previous study (Zhang et al., 2011). The degree ($S_{i}^{w}$) was defined as the sum of the weights of all the connections of node *i*, that is $S_{i}^{w}=\sum_{j\in N}W_{\mathrm{ij}}$. $S_{i}^{w}$ can be considered as centers for information integration. The degree $S_{i}^{w}$ measures the extent to which a node is relevant to the graph (Rubinov & Sporns, 2010). The total connection strength $S_{\mathrm{net}}^{w}$ of the network was calculated as the sum of $S_{i}^{w}$ for all nodes *N* in the network: $\;S_{\mathrm{net}}^{w}=\frac{1}{N}\sum_{j\in N}S_{i}^{w}$. The nodal efficiency of a given node *i* ($E_{i}^{w}$) is calculated as the inverse of the mean harmonic shortest path length between this node and all other nodes in the network (Achard, Salvador, Whitcher, Suckling, & Bullmore, 2006; Shu et al., 2011), according to the formula:

$$E_{i}^{w}=\frac{1}{N-1}\sum_{j\neq j\in G}\frac{1}{L_{\mathrm{ij}}}$$

where *L*_*ij*_ is the weighted shortest path length between nodes *i* and *j* in the network. $E_{i}^{w}$ quantifies the importance of the nodes for the communication within the network (Bassett & Bullmore, 2006). The betweenness centrality $B_{i}^{w}$ of a node *i* describes the fraction of all shortest paths in the network that pass through the node. In the present study, we calculated the normalized betweenness as $b_{i}^{w}$ = $B_{i}^{w}$/〈$B_{i}^{w}〉$, where 〈$B_{i}^{w}〉$ is the averaged nodal betweenness of the network. Nodes with high betweenness centrality may serve as way stations for network traffic.

The coupling between functional and structural connectivity networks for each subject was quantified as described in the previous study (Zhang et al., 2011). For each subject, we quantified the coupling between functional and structural connectivity networks. The correlation between functional and structural connectivity was constrained by the edges with non‐zeros structural connectivity. Specifically, the non‐zero structural connectivity network edges were extracted to produce a vector of structural connectivity values. Next, these values were resampled into a Gaussian distribution (Hagmann et al., 2010; Honey et al., 2009). The corresponding functional connectivity network edges were also extracted to form a vector of functional connectivity values. Subsequently, the coupling between functional and structural connectivity network was calculated by the Pearson's correlation between these two vectors.

### 2.8 Statistical analysis

Statistical analysis was performed using the software SPSS version 17.0 (SPSS Inc., Chicago, IL) for demographic and clinical data, and Gretna (http://www.nitrc.org/projects/gretna/) for fMRI data. A two‐way repeated measures ANOVA test was performed to examine the group‐by‐time interaction and the main effects of group and time in graph characteristics and nodal properties differences between the patient group and HC group over time. When interaction and main effect terms were significant in a brain region, the values of graph characteristics and nodal properties were extracted for additional post hoc comparisons, with two‐sample and repeated measures ANOVA tests as appropriate. The results were corrected using the false‐positive adjustment, setting a threshold at *p* < 1/90 = .011 (Jao et al., 2013). We correlated the change of clinical variables to change of the strength of functional–structural connectivity network coupling with Pearson's correlation analysis, setting a threshold at *p* < .05.

### 2.9 Reproducibility analysis

We conducted a split‐half analysis to test the reproducibility of our findings as described in the previous studies (He et al., 2009; Shu et al., 2011; Zhang et al., 2011). HC group was divided into six subgroups, matched for age, gender and education levels (two at each time point, Table S2). Similarly, the patient group was also divided into six matched subgroups (two at each time point, Table S2) (all *p* > .05). For each subgroup, both functional and structural connectivity networks were constructed and analyzed analogously to the aforementioned whole‐group analysis. Pearson's correlation coefficients for the correlation patterns of the both functional and structural connectivity networks between subgroups were calculated to test whether there was a consistent topological organization in the population (He et al., 2009). The topological parameters ($S_{\mathrm{net}}^{w}$ and $L_{\mathrm{net}}^{w}$) between each pair of subgroups were compared using permutation testing.

## 3 RESULTS

### 3.1 Demographics and behavioral tests

There were no differences in gender (*p* = .224) and age (*p* = .383) between ESRD patients and HC. A group‐by‐time interaction effect was identified in the SDS scores (*p* = .007) (Table 1). Post hoc analyses revealed that ESRD patients before KT had significantly higher SDS scores than HC at baseline (*p* = .009) (Table 1). At one and 6‐month follow‐up, all these abnormalities recovered toward normal levels relative to HC (All *p* > .05) (Table 1). In addition, significant main effects of time were found in the SDS scores for the patient group (*p* = 0.007) (Table 1), suggesting improved mood levels.

**Table 1:** Demographics, clinical characteristics, and cognitive performance of patients and healthy controls

Protocols	Groups	Before or after kidney transplantation			*F* value	*p* value
		Baseline	1‐month	6‐month		
NCT (s)	HC	25.2 ± 5.0	24.4 ± 6.2	23.9 ± 5.4	0.478	.625^c^
	Patients	42.0 ± 17.8	39.3 ± 14.6	39.4 ± 20.7	0.860	.431^c^
	*p* value	**<.001** b ^,^ *	**<.001** b ^,^ *	**.003** b ^,^ *	*F* = 0.209, *p* = .812^d^	
DST (score)	HC	69.1 ± 6.3	75.4 ± 7.4	77.8 ± 8.6	15.920	**<.001** c ^,^ *
	Patients	54.2 ± 12.8	57.8 ± 12.8	62.0 ± 12.7	17.172	**<.001** c ^,^ *
	*p* value	**<.001** b ^,^ *	**<.001** b ^,^ *	**<.001** b ^,^ *	*F* = 0.939, *p* = .396^d^	
LTT (s)	HC	30.1 ± 11.4	34.0 ± 10.9	30.0 ± 8.7	2.628	.088^c^
	Patients	53.4 ± 10.0	53.1 ± 11.6	49.0 ± 9.9	1.155	.325^c^
	*p* value	**<.001** b ^,^ *	**<.001** b ^,^ *	**<.001** b ^,^ *	*F* = 0.728, *p* = .487^d^	
SDT (s)	HC	29.0 ± 5.5	28.8 ± 5.4	28.4 ± .3	0.203	.817^c^
	Patients	48.5 ± 9.0	49.3 ± 8.8	48.1 ± 12.9	0.100	.905^c^
	*p* value	**<.001** b ^,^ *	**<.001** b ^,^ *	**<.001** b ^,^ *	*F* = 0.053, *p* = .948^d^	
SDS (score)	HC	26.6 ± 4.9	27.4 ± 5.5	27.0 ± 5.6	1.018	.385^c^
	Patients	34.4 ± 11.6	27.1 ± 6.3	27.8 ± 8.0	6.637	**.007** c ^,^ *
	*p* value	**.009** b ^,^ *	.891^b^	.737^b^	*F* = 5.724, *p* = **.007** d ^,^ *	
BUN (mg/dl)	HC	13.4 ± 3.0	12.5 ± 2.6	13.3 ± 1.8	1.279	.292^c^
	Patients	67.1 ± 22.6	20.6 ± 6.9	18.9 ± 5.6	48.469	.000^c^
	*p* value	**<.001** b ^,^ *	**<.001** b ^,^ *	**<.001** b ^,^ *	*F* = 40.810, ***p* = .000** d	
Scr (mg/dl)	HC	0.7 ± 0.2	0.7 ± 0.2	0.7 ± 0.2	0.285	.754^c^
	Patients	11.6 ± 2.8	1.2 ± 0.5	1.2 ± 0.3	136.190	**<.001** c
	*p* value	**<.001** b ^,^ *	**<.001** b ^,^ *	**<.001** b ^,^ *	*F* = 111.797, ***p* < .001** d	
RBC count (×10^12^)	HC	4.7 ± 0.5	4.7 ± 0.6	5.1 ± 0.6	7.565	**.005** c
	Patients	3.6 ± 0.7	4.1 ± 0.4	4.6 ± 0.7	25.940	**.000** c ^,^ *
	*p* value	**<.001** b ^,^ *	**.001** b ^,^ *	**.032** b ^,^ *	*F* = 8.200, *p* = .001^d^^,^ *	
Hemoglobin (g/L)	HC	141.5 ± 16.7	139.2 ± 18.0	151.7 ± 19.3	9.857	**.000** c
	Patients	108.3 ± 22.0	125.6 ± 14.2	138.1 ± 18.4	21.089	**.000** c
	*p* value	**<.001** b ^,^ *	**.013** b ^,^ *	**.034** b ^,^ *	*F* = 7.617, ***p* = .001** d	
Hematocrit (L/L)	HC	0.4 ± 0.0	0.4 ± 0.0	0.4 ± 0.1	6.942	**.007** c ^,^ *
	Patients	0.3 ± 0.1	0.4 ± 0.0	0.4 ± 0.1	20.248	**.000** c ^,^ *
	*p* value	**<.001** b ^,^ *	.167^b^	.304^b^	*F* = 7.1000, ***p* = .002** d ^,^ *	

*Note*: Continuous data are expressed as mean and *SD*. Statistical threshold is set at *p* < .05.

Abbreviations: BUN, blood urea nitrogen; DST, digital symbol test; HC, healthy controls; KT‐1m, 1‐month after kidney transplantation; KT‐6m, 6‐month after kidney transplantation; LTT, line‐tracing test; NCT‐A, number connection test‐A; Pre‐KT, pre‐kidney transplantation; RBC, red blood cells; SAS, Zung self‐rating anxiety scale; SDS, Zung self‐rating depression scale; SDT, serial‐dotting test. Less time in NCT‐A, LTT, SDT and lower scores on SAS and SDS reflect better performance, while higher DST scores indicate better performance.

a The *p* values are obtained by using the *χ* ^2^ test.

b The *p* values are obtained by using a two‐sample *t* test; data in parentheses are 95% confidence interval.

c The *p* values are obtained by using a one way repeated measures ANOVA test.

d The *p* values are obtained by using a two‐way repeated measures ANOVA.

*Statistically significant difference.

In addition, significant main effects of group were found in NCT‐A, DST, LTT, and SDT scores. Post hoc analyses revealed that patients had significantly higher NCT‐A, LTT, SDT scores and lower DST scores at all three time points (All *p* < .05) (Table 1), indicating worse performance in the patient group. Significant main effects of time were found in the DST scores (*p* < .001) (Table 1). With time elapsing, both HC and patients showed an increasing trend in DST scores (All *p* < .05) (Table 1).

### 3.2 Clinical data

After KT, the urea, serum creatinine, and uric acid decreased significantly compared with pre‐KT (All *p* < .05) while hemoglobin, hematocrit, and red blood cell count increased significantly (All *p* < .05) (Table 1). No significant difference was found in hematocrit level between the patients and HC at 6 months after KT (*p* = .304).

### 3.3 Overall topology of functional and structural connectivity networks

Both ESRD patients and HC showed a small‐world organization in functional and structural connectivity networks constructed at all connection densities (functional: 0.05–0.20; structural: 0.05–0.15), following the AAL‐90 parcellation scheme. There were no significant differences (all *p* > .05) for the functional and structural connectivity networks between two groups.

### 3.4 Nodal characteristics

#### 3.4.1 KT‐related functional connectivity alterations

##### *S*_*i*_ change pattern

**Figure 1:**
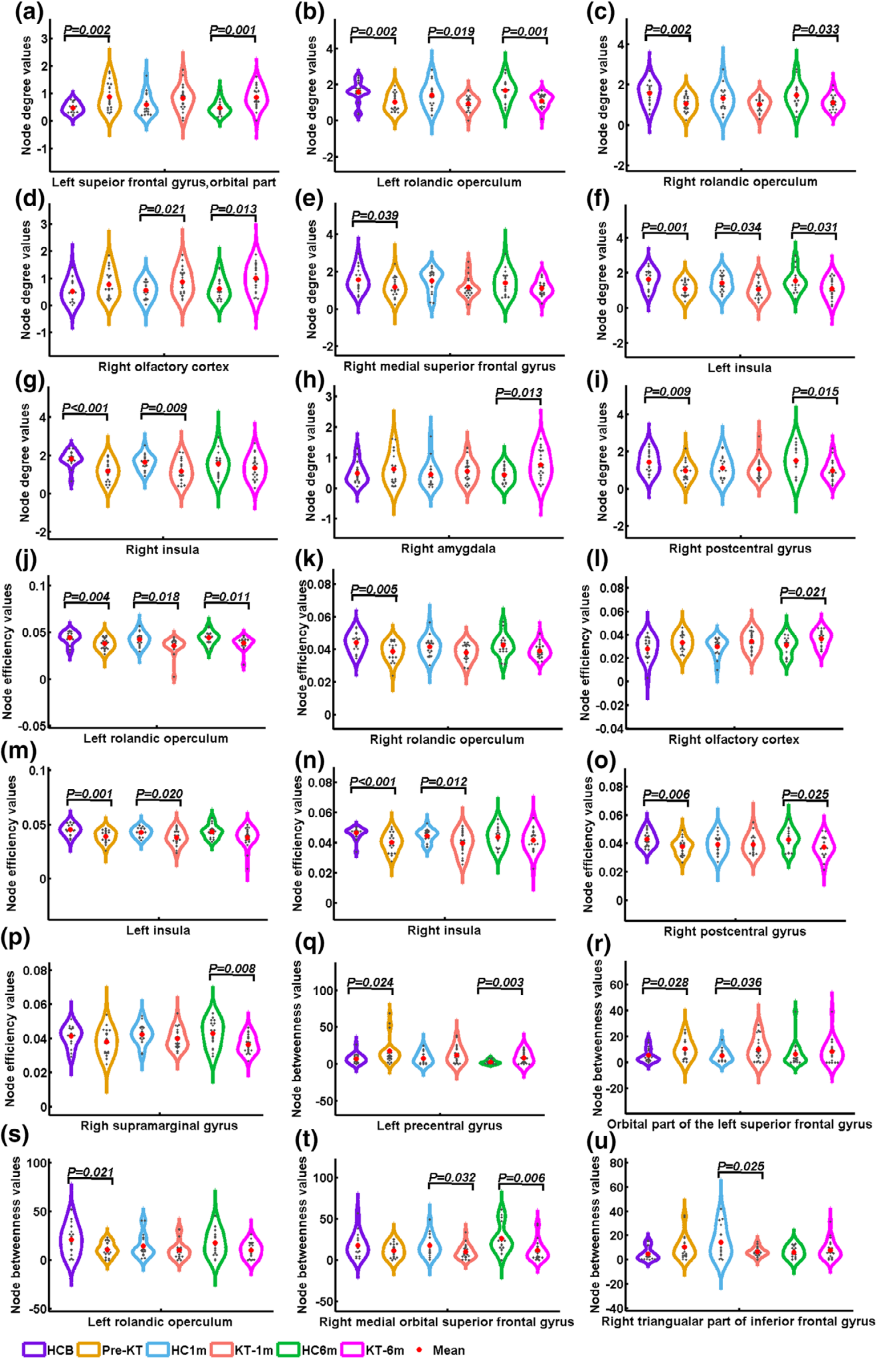
Alterations of node degree, node efficiency, and node betweenness of functional connectivity networks in patients before and after kidney transplantation relative to healthy controls. The plots showing the significant main effect of group in node degree of functional connectivity networks in the left orbital part of superior frontal gyrus, bilateral rolandic operculum, bilateral insula, right olfactory cortex, right medial superior frontal gyrus, right amygdala and right postcentral gyrus (Panels a–i). Significant main effects of group for node efficiency of the functional connectivity in bilateral rolandic operculum, bilateral insula, right olfactory cortex, right postcentral gyrus, and right supramarginal gyrus (Panels j–p). Significant main effects of group were found in *B*_*i*_ of left precentral gyrus, orbital part of the left superior frontal gyrus, left rolandic operculum, and right medial orbital superior frontal gyrus (Panels q–t). The group‐by‐time interaction effect on *B*_*i*_ values in the right triangular part of inferior frontal gyrus (Panel u). HCB, healthy control at baseline; HC1m, healthy control at 1‐month follow‐up; HC6m, healthy control at 6‐month follow‐up; KT‐1m, 1‐month after kidney transplantation; KT‐6m, 6‐month after kidney transplantation; pre‐KT, pre‐kidney transplantation [Color figure can be viewed at http://wileyonlinelibrary.com]

Significant main effects of group were found in *S*_*i*_ of the left orbital part of superior frontal gyrus, bilateral rolandic operculum, bilateral insula, right olfactory cortex, right medial superior frontal gyrus, right amygdala, and right postcentral gyrus (Figure 1). Post hoc analyses indicated that patients had higher *S*_*i*_ values in the left orbital part of superior frontal gyrus before (*p* = .002) and 6‐month (*p* = .001) after KT compared with HC group. Patients had lower *S*_*i*_ values in the left rolandic operculum and left insula compared with HC group at all three time points (All *p* < .05). Compared with HC group, patients had lower *S*_*i*_ values in the right rolandic operculum and right postcentral gyrus before and 6 months after KT (All *p* < .05). For the right olfactory cortex, patients had higher *S*_*i*_ values 1 month (*p* = .021) and 6 months (*p* = .013) after KT. Pre‐KT patients had lower *S*_*i*_ values in the right medial superior frontal gyrus (*p* = .039). For the right insula, patients before (*p* < .001) and 1 month (*p* = .009) after KT had decreased *S*_*i*_ values. Patients had higher *S*_*i*_ values in the right amygdala (*p* = .013) 6 months after KT.

##### *E*_*i*_ change pattern

Significant main effects of group were found in *E*_*i*_ of bilateral rolandic operculum, bilateral insula, right olfactory cortex, right postcentral gyrus and right supramarginal gyrus. Post hoc analyses indicated that patients had lower *E*_*i*_ values in the left rolandic operculum and left insula compared with HC group at all three time points (All *p* < .05). Compared with HC group, patients had lower *E*_*i*_ values in the right rolandic operculum (*p* = .005). For the right olfactory cortex, patients had higher *E*_*i*_ values 6 months (*p* = .021) after KT. For the right insula, patients before (*p* < .001) and 1 month (*p* = .012) after KT had decreased *E*_*i*_ values. Compared with HC group, patients had lower *E*_*i*_ values in the right postcentral gyrus pre‐KT (*p* = .006) and 6 months (*p* = .025) after KT. Patients had decreased *E*_*i*_ values in the right supramarginal gyrus (*p* = .008) 6 months after KT.

##### *B*_*i*_ change pattern

A group‐by‐time interaction effect on *B*_*i*_ values was identified in the right triangular part of inferior frontal gyrus. Post hoc analyses revealed that patients 1‐month after KT had lower *B*_*i*_ than HC group (*p* = .025). Significant main effects of group were found in *B*_*i*_ of left precentral gyrus, orbital part of the left superior frontal gyrus, left rolandic operculum, and right medial orbital superior frontal gyrus. Post hoc analyses revealed that patients had higher *B*_*i*_ values in the left precentral gyrus before (*p* = .024) and 6 months (*p* = .003) after KT. Compared with HC group, patients before (*p* = .028) and 1 month (*p* = .036) after KT had higher *B*_*i*_ values in orbital part of the left superior frontal gyrus. Patients had lower *B*_*i*_ values in the left rolandic operculum before KT (*p* = .021). Patients had lower *B*_*i*_ values in the right medial orbital superior frontal gyrus one and 6 months after KT (*p* = .032; *p* = .006).

#### 3.4.2 KT‐related structural connectivity alterations

##### *S*_*i*_ change pattern

**Figure 2:**
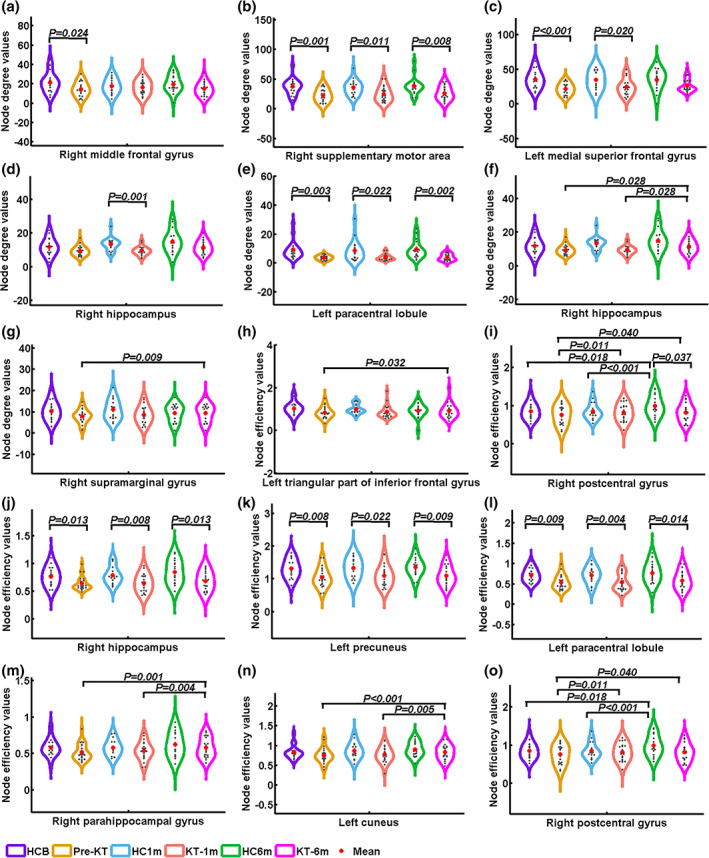
Alterations of node degree, node efficiency of structural connectivity networks in patients before and after kidney transplantation relative to healthy controls. The plots showing a group‐by‐time interaction effect on node degree values in the structural connectivity network in the right middle frontal gyrus (Panel a). Significant main effects of group were found in node degree of the right supplementary motor area, right hippocampus, left medial superior frontal gyrus, and left paracentral lobule (Panels b–e). A group‐by‐time interaction effect on node efficiency values was found in the structural connectivity network in the left triangular part of inferior frontal gyrus and right postcentral gyrus (Panels h and i). Significant main effects of group were found in node efficiency of right hippocampus, right paracentral lobule and left precuneus (Panels j–l). Significant main effects of time of node efficiency were found in the right parahippocampal gyrus, left cuneus, and right postcentral gyrus (Panels m–o). HCB, healthy control at baseline; HC1m, healthy control at 1‐month follow‐up; HC6m, healthy control at 6‐month follow‐up; KT‐1m, 1‐month after kidney transplantation; KT‐6m, 6‐month after kidney transplantation; pre‐KT, pre‐kidney transplantation [Color figure can be viewed at http://wileyonlinelibrary.com]

In the structural connectivity network, a group‐by‐time interaction effect on *S*_*i*_ values was identified in the right middle frontal gyrus (Figure 2). Post hoc analyses revealed that patients before KT had lower *S*_*i*_ in the right middle frontal gyrus than HC group (*p* = .024). Significant main effects of group were found in *S*_*i*_ of right supplementary motor area, right hippocampus, left medial superior frontal gyrus and left paracentral lobule. Post hoc analyses revealed that patients had significantly decreased *S*_*i*_ in the right supplementary motor area and left paracentral lobule than HC at all three time points (All *p* < .05). For the left medial superior frontal gyrus, patients had significantly decreased *S*_*i*_ before and 1‐month after KT. Decreased *S*_*i*_ was found in the KT‐1m group. Significant main effects of time were found in *S*_*i*_ of right hippocampus and right supramarginal gyrus. Post hoc analyses revealed that pre‐KT (*p* = .028) and KT‐1m (*p* = .028) patients had significantly lower *S*_*i*_ in the right hippocampus than HC. The patient group showed an increasing trend in *S*_*i*_ in the right supramarginal gyrus. Specifically, pre‐KT patients had lower *S*_*i*_ for the right supramarginal gyrus than the KT‐6m group (*p* = .009).

##### *E*_*i*_ change pattern

A group‐by‐time interaction effect on *E*_*i*_ values was identified in the left triangular part of inferior frontal gyrus and right postcentral gyrus. Post hoc analyses revealed that pre‐KT patients had lower *E*_*i*_ in the left triangular part of inferior frontal gyrus than the KT‐6m group (*p* = .032). KT‐6m group had lower *E*_*i*_ for the right postcentral gyrus than HC (*p* = .037). Significant main effects of group were found in *E*_*i*_ of the right hippocampus, right paracentral lobule, and left precuneus. Post hoc analyses revealed that pre‐KT and KT‐1m (*p* = .028) patients had significantly lower *E*_*i*_ in the right hippocampus, right paracentral lobule, and left precuneus (All *p* < .05). Significant main effects of time were found in *E*_*i*_ of the right parahippocampal gyrus, left cuneus, and right postcentral gyrus. Post hoc analyses revealed that pre‐KT (*p* = .028) and KT‐1m (*p* = .028) patients had significantly lower *E*_*i*_ in the right parahippocampal gyrus and left cuneus than HC. Both the patients and HC at baseline and 1 month follow‐up had lower *E*_*i*_ for the right postcentral gyrus than the 6 months follow‐up (All *p* < .05).

### 3.5 Edge characteristics

**Figure 3:**
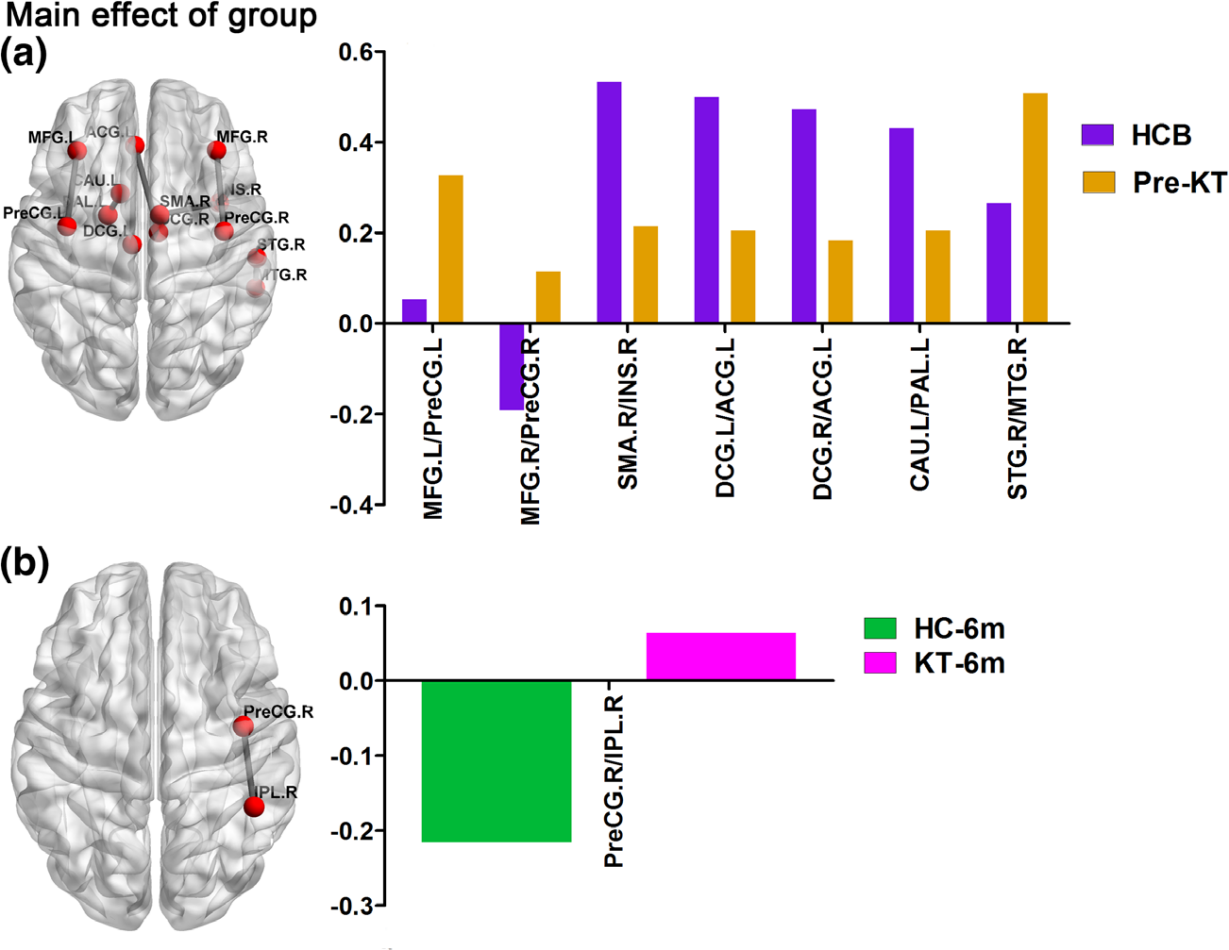
Functional connectivity network before and after kidney transplantation. Pre‐KT patients had seven altered functional connectivities compared with the HCB (Panel a), while KT‐6m patients had one increased negative functional connectivity compared with the HC6m group (Panel b). HCB, healthy control at baseline; HC6m, healthy control at 6‐month follow‐up; KT‐6m, 6‐month after kidney transplantation; pre‐KT, pre‐kidney transplantation [Color figure can be viewed at http://wileyonlinelibrary.com]

For the functional connectivity network, a group‐by‐time interaction effect or main effect of group on functional connectivity network values was identified in the 26 connectivities. Post hoc analyses revealed that pre‐KT patients had seven altered functional connectivities compared with HC group at baseline (Figure 3a). KT‐1m patients showed no altered functional connectivity compared with the 1 month follow up HC group. KT‐6m patients had one increased negative functional connectivity compared with the 6 months follow up HC group (Figure 3b).

**Figure 4:**
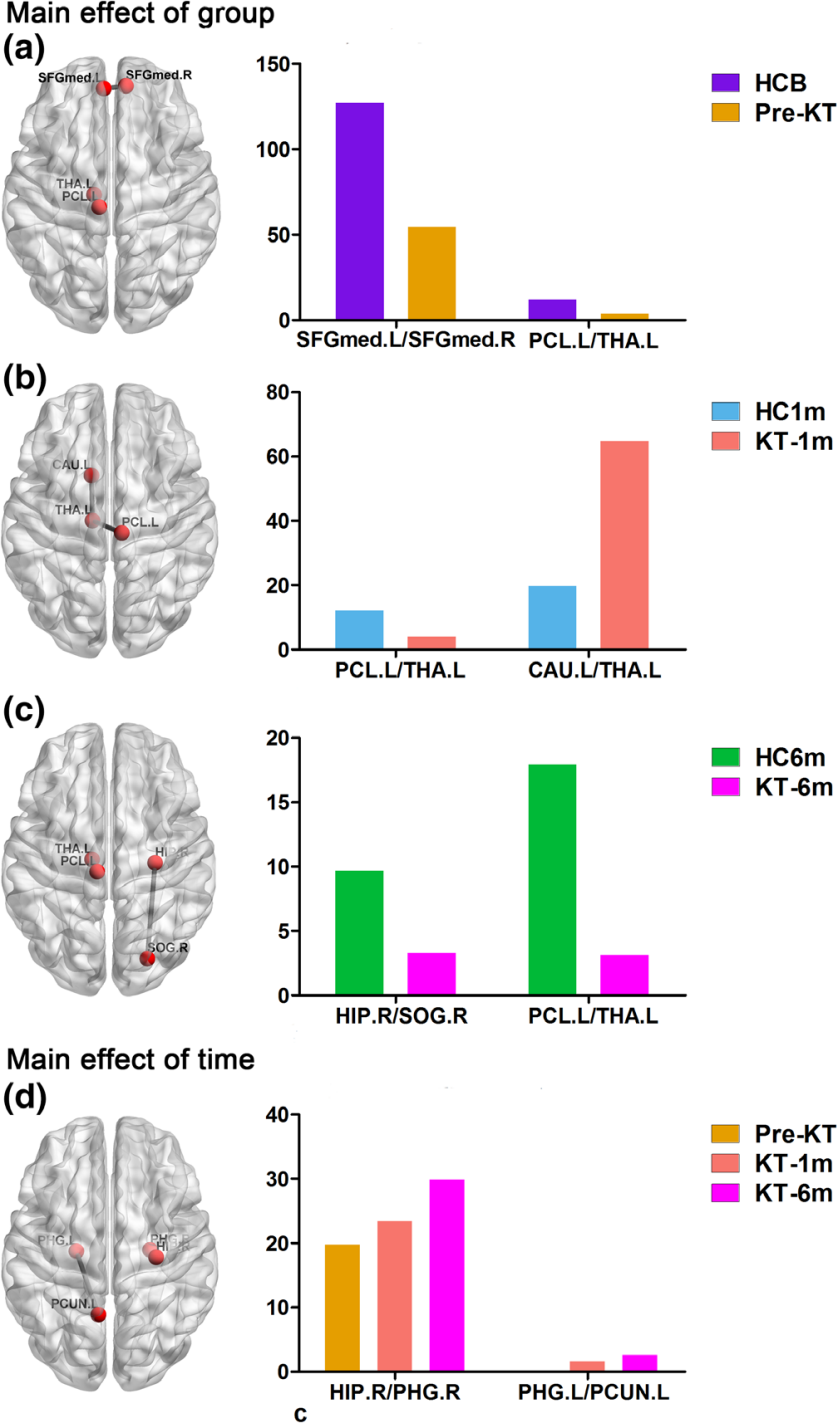
Structural connectivity network before and after kidney transplantation. For the structural connectivity network, pre‐KT patients showed two decreased positive functional connectivities compared with the HCB (Panel a). KT‐1m patients had one decreased and one increased positive functional connectivities compared with the HC1m group (Panel b). KT‐6m patients had two decreased positive functional connectivities compared with the HC6m group (Panel c). For the patient group, a group‐by‐time interaction effect was identified in two connectivities. Patients after KT showed an increasing trend in these two connectivities (Panel d). HCB, healthy control at baseline; HC1m, healthy control at 1‐month follow‐up; HC6m, healthy control at 6‐month follow‐up; KT‐1m, 1‐month after kidney transplantation; KT‐6m, 6‐month after kidney transplantation; pre‐KT, pre‐kidney transplantation [Color figure can be viewed at http://wileyonlinelibrary.com]

For the structural connectivity network, a group‐by‐time interaction effect or main effect of group on structural connectivity network values was identified in the seven connectivities. Post hoc analyses revealed that pre‐KT patients had two decreased positive functional connectivities compared with HC group at baseline (Figure 4a). KT‐1m patients had one decreased and one increased positive functional connectivities compared with the 1 month follow up HC group (Figure 4b). KT‐6m patients had two decreased positive functional connectivities compared with the 6 months follow up HC group (Figure 4c). For the patient group, a group‐by‐time interaction effect was identified in two connectivities. Patients after KT showed an increasing trend in the two connectivities (Figure 4d).

### 3.6 Altered functional–structural connectivity coupling

**Figure 5:**
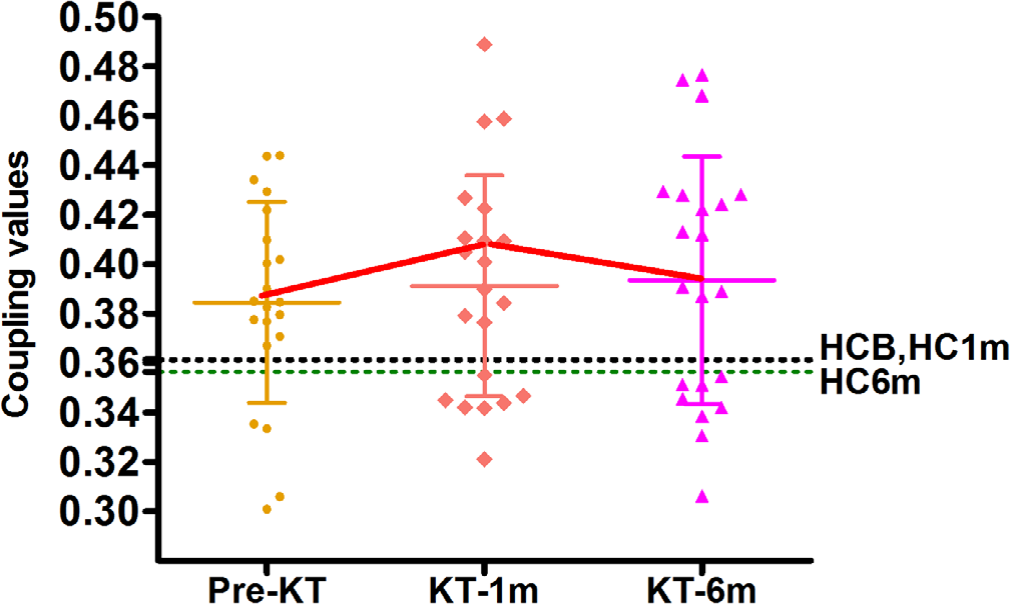
The average functional–structural connectivity coupling of the patients and healthy controls. The patients showed an increase in strength of functional–structural connectivity coupling at 1 month after kidney transplantation. HCB, healthy control at baseline; HC1m, healthy control at 1‐month follow‐up; HC6m, healthy control at 6‐month follow up; KT‐1m, 1‐month after kidney transplantation; KT‐6m, 6‐month after kidney transplantation; pre‐KT, pre‐kidney transplantation [Color figure can be viewed at http://wileyonlinelibrary.com]

**Figure 6:**
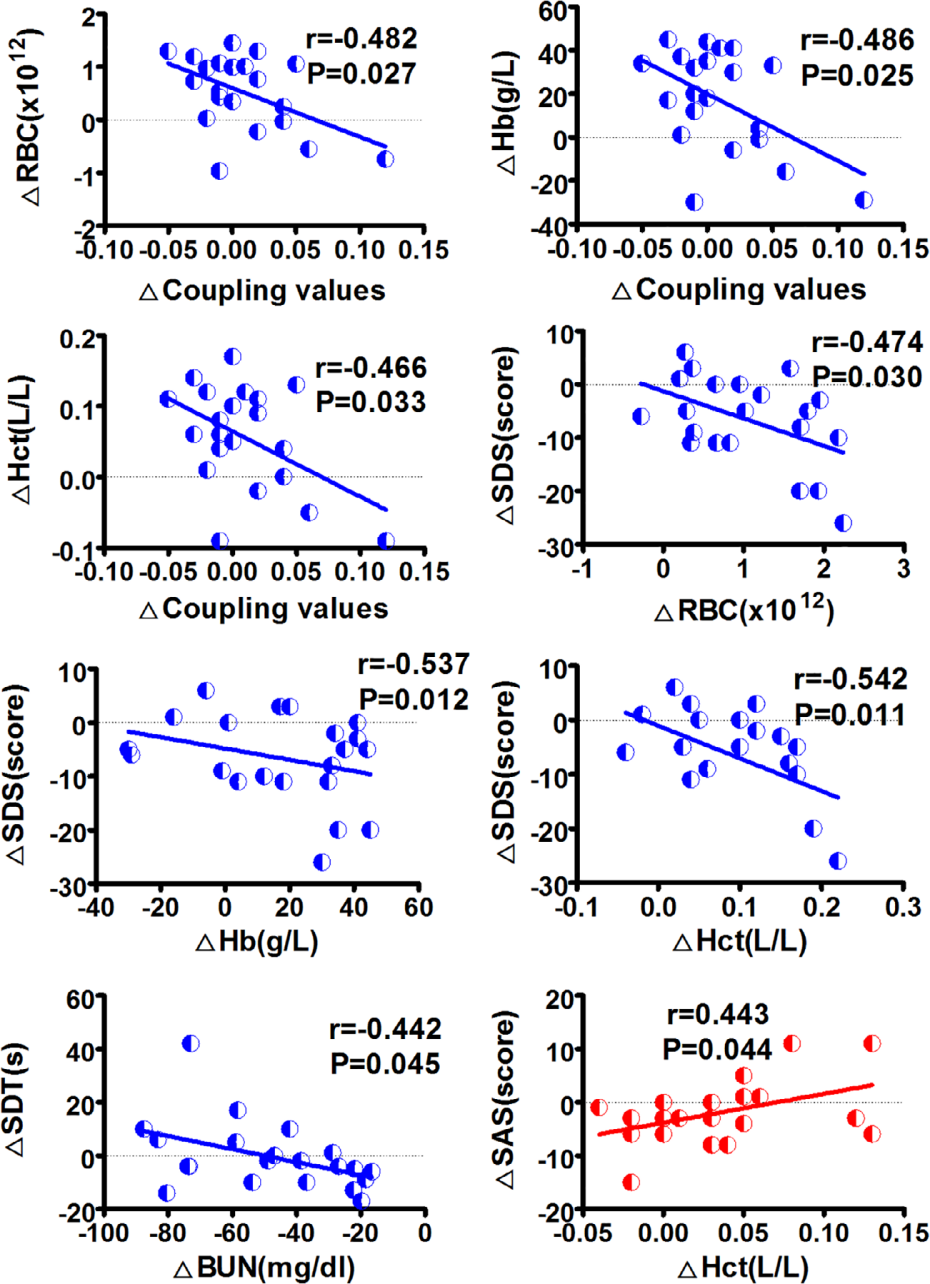
Correlation results. Change in the strength of functional–structural connectivity coupling negatively correlated with change in red blood cell (RBC), hemoglobin (Hb), and hematocrit (Hct) during the pre‐KT and 1‐month follow‐up. Change in SDS scores was negatively correlated with change in RBC, Hb, and Hct during the pre‐KT and 6‐month follow‐up. Change in SDT scores was negatively correlated with change in blood urea nitrogen (BUN) during the pre‐KT and 6‐month follow‐up. Change in SAS scores was positively correlated with change in Hct during the pre‐KT and 6‐month follow‐up. KT‐1m, 1‐month after kidney transplantation; KT‐6m, 6‐month after kidney transplantation; pre‐KT, pre‐kidney transplantation; SAS, Zung self‐rating anxiety scales; SDS, Zung self‐rating depression scale; SDT, serial dotting test [Color figure can be viewed at http://wileyonlinelibrary.com]

Figure 5 shows functional–structural connectivity coupling of each patient before, 1 month and 6 months after KT. Significant main effects of group was found for the coupling values (*p* = .041). Post hoc analyses revealed that KT‐1m patients had higher coupling values than HC at 1‐month follow up (*p* = .041). No difference was found for the coupling between patients at all three time points (All *p* > .05). Moreover, change in the strength of functional–structural connectivity coupling negatively correlated with change in red blood cell (*r* = −.482, *p* = .027), hemoglobin (*r* = −.486, *p* = .025), hematocrit (*r* = −.466, *p* = .033) during the pre‐KT and KT‐1m (Figure 6). Change in SDS scores was negatively correlated with change in red blood cell (*r* = −.474, *p* = .030), hemoglobin (*r* = −.537, *p* = .012), hematocrit (*r* = −.542, *p* = .011) during the pre‐KT and KT‐6m (Figure 6). Change in SDT scores was negatively correlated with change in blood urea nitrogen (*r* = −.442, *p* = .045) during the pre‐KT and KT‐6m follow up (Figure 6). Change in SAS scores was positively correlated with change in hematocrit (*r* = .443, *p* = .044) during the pre‐KT and KT‐6m follow up (Figure 6).

### 3.7 Reproducibility results

**Figure 7:**
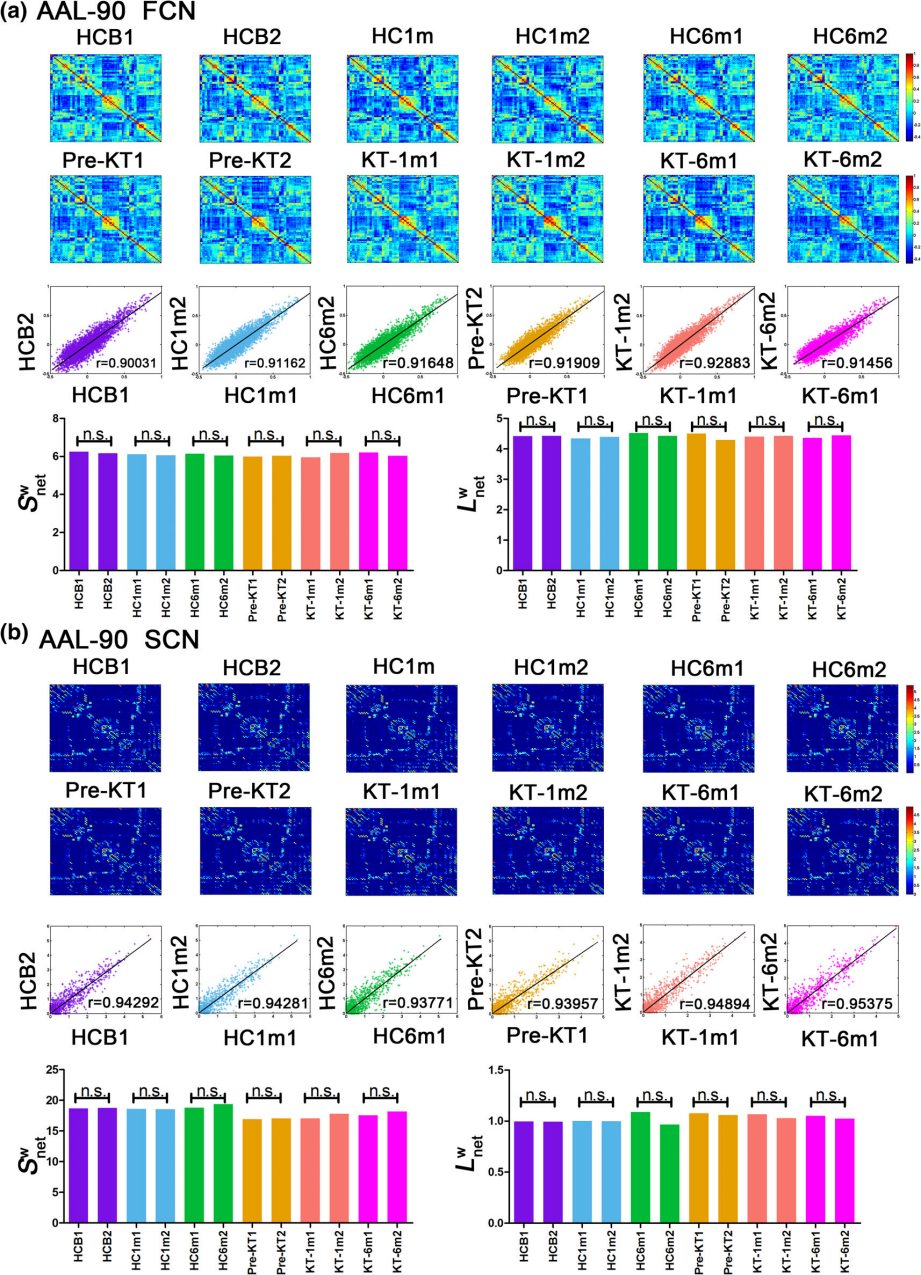
Evaluation of the reproducibility of the results for the functional connectivity network (Panel a) and structural connectivity network (Panel b). First and second row: mean network matrices of each subgroup (HCB1, HCB2, HC1m1, HC1m2, HC6m1, HC6m2, pre‐KT1, pre‐KT2, KT‐1m1, KT‐1m2, KT‐6m1, and KT‐6m2); the edge represents the connection weight between nodes. (First to third row): the correlation between two healthy control subgroups and between the patient subgroups. (Bottom row): No significant differences were shown in the two nodal characteristics between the two healthy control subgroups and between the patient subgroups. FCN, functional connectivity network; HCB, healthy control at baseline; HC1m, healthy control at 1‐month follow‐up; HC6m, healthy control at 6‐month follow up; KT‐1m, 1‐month after kidney transplantation; KT‐6m, 6‐month after kidney transplantation; pre‐KT, pre‐kidney transplantation; SCN, structural connectivity network [Color figure can be viewed at http://wileyonlinelibrary.com]

A significant correlation in weighted networks was observed when comparing the six HC subgroups (HCbaseline: *r* = .900 and *r* = .957; HC1m: *r* = .912 and *r* = .943; HC6m: *r* = .916 and *r* = .938 for AAL‐90 functional and structural connectivity networks, respectively). A finding was also seen for the six subgroups (pre‐KT: *r* = .919 and *r* = .940; KT‐1m: *r* = .929 and *r* = .949; KT‐6m: *r* = .915 and *r* = .954 for AAL‐90 functional and structural connectivity networks, respectively) (Figure 7). Neither comparison between the six HC subgroups (Figure 7, *p* > .05) nor comparison between six subgroups (*p* > .05) showed significant differences in $S_{\mathrm{net}}^{w}$ and $L_{\mathrm{net}}^{w}$. When compared patients with HC groups, significant differences were observed in node degree and node efficiency for the functional (Figure S1) and structural connectivity networks (Figure S2). Neither comparison between the HC subgroups (*p* < .05) nor comparison between the patient subgroups showed significant differences in coupling values, significant difference was found between the HC subgroups and patients' subgroups (Figure S3). These results suggest a high reproducibility of our findings.

## 4 DISCUSSION

The results of the present study showed a progressive recovery trend in patients first 6 months after successful KT. However, functional and structural networks did not recover to the normal levels even 6 months after KT. This suggests that fMRI might be a useful tool to interpret some clinical phenomenon.

### 4.1 Altered nodal topology of functional and structural connectivity networks

An important finding of this study is that a progressive recovery trend was observed in patients after KT. This is particularly prominent in the structural changes of *S*_*i*_. One previous study found that the cognitive benefits of KT continued to develop over time (Harciarek et al., 2011). However, they did not elucidate the pathophysiological mechanism. Our study could obtain more information of altered brain activity underlying KT by combining both functional and structural methods of brain activity. As expected, the *S*_*i*_ and *E*_*i*_ of structural network seemed to need more time to recover than those in the functional network. This is consistent with our previous study that functional network may recover earlier than structural connectivity changes after KT (Zhang et al., 2016). It appears that the recovery of functional and structural network was not synchronous. However, more studies are further needed to explain the phenomenon.

Several previous studies have found the abnormalities in DMN regions in ESRD patients (Luo et al., 2016; Ni et al., 2014). However, there is no investigation of the effect of KT on the complex brain network in the ESRD patients at the topology level. In this study, we found abnormal *S*_*i*_ and *E*_*i*_ in DMN regions such as insula, hippocampus and precuneus in pre‐KT patient group. Dysfunction of these regions might be associated with cognitive impairment in the ESRD patients. As expected, these domains particularly in the structural network did not recover to the normal levels even 6 months after KT. This is in line with the clinical observations that some domains of cognitive function did not recover completely at the early stage of KT. This is particularly true in memory function (Harciarek et al., 2011). It seems that memory problems are only indirectly related to the effects of chronic kidney disease related neurotoxicity (Harciarek et al., 2011).

### 4.2 Altered functional and structural connectivity networks

In this study, functional connectivity changes in the patient groups were not uniform, while structural connectivity was uniformly decreased compared with HC group. Generally speaking, the functional connectivity is regarded to be more flexible; while the structural connectivity network remains relatively stable (Bullmore & Sporns, 2009). Thus, the alterations of functional connectivity may be more complex than structural connectivity. The pre‐KT patient showed weaker structural connectivity between the left paracentral lobule and left thalamus. Thalamus plays an important role in neural input and output (Tamminga & Buchsbaum, 2004). The weaker structural connectivity between the two brain regions might possibly result in memory problems. Interestingly, the patients showed an increased structural connectivity in hippocampus‐parahippocampus, parahippocampus‐precuneus after KT, suggesting a gradual recovery trend observed in these patients. The hippocampus is necessary for memory functions, especially memorizing facts and events (Braun, 2011). The precuneus mainly involves in memory encoding, consolidation, and environmental monitoring. Disruption to the hippocampus and precuneus connectivity leads to the inability to form new memories (Braun, 2011). Increased connectivity between the hippocampus and the parahippocampus means memory function is recovering.

### 4.3 Decoupling between functional and structural connectivity networks

In this study, we investigated the structure–function relations in large‐scale brain networks in patients after KT for the first time. It is currently accepted that structural connections are highly predictive of the presence and strength of functional connections and invariably inform and constrain biological function (Honey, Thivierge, & Sporns, 2010). However, structural connections cannot be inferred reliably on the basis of observed functional coupling, since strong functional connections may also exist between the regions that are not directly anatomically linked (Honey et al., 2010). Functional connectivity fluctuates in a complex pattern, reflective of the rich underlying dynamics. As expected, there was aberrant functional–structural connectivity coupling in the patients. An increased network coupling between functional and structural connectivity was found in the patients within 1 month after KT compared with HC at 1‐month follow‐up. We deduced that the patients might experience the most dramatical change 1 month after KT. Therefore, this is of clinical implication that more attention should be paid to the patients at 1 month after KT. Additionally, the correlation between changes in the strength of functional–structural connectivity and red blood cell, hemoglobin and hematocrit further highlighted the importance of treating anemia in ESRD patients (Luo et al., 2016). Improving these anemia‐related parameters may possibly result in cognition improvement.

### 4.4 Limitations

This study has some limitations. Firstly, the sample size is small and not all the patients received the same treatment before KT. Secondly, 6‐month follow‐up may be insufficient to observe all potential benefits of KT for brain structure and function. Thirdly, the perioperative insult and the neural toxicity of immunosuppressant drugs may have an adverse influence on the cognitive function recovery in patients receiving KT. Fourthly, the same scales were used to assess cognitive status in our follow‐up studies and learning effects could not be avoided. Last but not least, fatigue is found to be associated with cognition in many diseases such as post‐stroke and multiple sclerosis (de Rodez Benavent et al., 2017; Lagogianni, Thomas, & Lincoln, 2018), but we did not evaluate the fatigue in ESRD patients in our study. More efforts should be made to clarify the relationship between cognition and fatigue in the future study.

## 5 CONCLUSIONS

In conclusion, abnormal nodal characteristics of functional and structural connectivity networks were found in ESRD patients. Importantly, a progressive recovery trend was observed in patients after KT but some DMN regions such as insula, hippocampus, and precuneus did not recover completely at the early stage following KT, which may provide novel insights into the mechanisms of cognitive improvement in KT patients. All in all, the present study demonstrates that the cognition improvement in KT patients is related to an improved topological organization in large‐scale brain structural and functional network, providing a new avenue for better understanding this disorder.

## CONFLICT OF INTEREST

All authors have no conflicts of interest to declare.

## Supporting information


**Appendix S1**: Supporting Information

## Data Availability

The datasets used during the current study are available from the corresponding author on reasonable request.
