# Supplementary material for: Functional–structural relationship in large‐scale brain networks of patients with end stage renal disease after kidney transplantation: A longitudinal study
Source: Hum Brain Mapp. 2019 Oct 1;41(2):328–41. doi: 10.1002/hbm.24804 (PMC7268055; doi:10.1002/hbm.24804)
Supplement: Supplementary file 1 — Appendix S1: Supporting Information [file HBM-41-328-s001.docx]

**Supplementary Materials:**

**Brain Network Construction**

**Functional Connectivity Network Construction**

Preprocessing of functional images was carried out using the Statistical Parametric Mapping software (SPM8, http://www.fil.ion.ucl.ac.uk/spm). The first 10 volumes of the functional images were discarded to eliminate the possible effects of imager instability and to allow adaptation of participants to the environment. The slice timing and head motion correction were performed. No participant was removed for the head motion greater than 1.0 mm or 1.0°. Then, spatial normalization was conducted to the standard Montreal Neurologic Institute (MNI) template, with a resampled voxel size of 3 × 3 × 3 mm^3^. No spatial smoothing was used to avoid introducing artificial local spatial correlations. For each subject, representative time series in each region of interest were obtained by averaging the functional MRI time series across all voxels in the region of interest. To reduce the effects of confounding factors unlikely to be involved in specific regional correlation, time series were preprocessed as follows: first, six head motion parameters, averaged signals from cerebrospinal fluid and white matter, and global brain signal were regressed (Fox, et al., 2005; Fox, et al., 2009); next, the time series were band-pass filtered (0.01–0.08 Hz). Correlation matrix of each patient was gained by using Gretna (http://www.nitrc.org/projects/gretna/). Automated Anatomic Labeling (AAL) template was used for the brain parcellation, only cortex areas were used in later analysis. A temporal correlation matrix (N×N, where N = 90 is the number of regions of interest in AAL-90 (Tzourio-Mazoyer, et al., 2002) was then obtained for each subject by computing Pearson correlation coefficients between the processed time series of **each** pair of regions of interest. A weighted network can incorporate additional information on the strength of functional connections on continuous scales, enabling more comprehensively understanding network organizations. To construct weighted functional connectivity networks, weighted edges were considered as absolute functional connectivity strength between connected regions of interest, e.g. w*_ij_* = | r*_ij_*|, where r*_ij_* is the correlation coefficient for nodes *_i_* and *_j_*.

**Structural Connectivity Network Construction**

For each subject, diffusion weighted images were eddy corrected by eddy tool in PANDA (Cui, et al., 2013). Diffusion tensor models were estimated by the linear least-squares fitting method at each voxel using the Diffusion Toolkit (http://trackvis.org). Whole-brain fiber tracking was performed in native diffusion space for each subject using the interpolated streamline algorithm embedded in the Diffusion Toolkit (Mori, et al., 1999). When the fractional anisotropy was < 0.15 or the tracing angle exceeded 35°, fiber tracing was stopped (Liao, et al., 2011). To determine the nodes of structural connectivity network in each subject, regions of interest were transformed into native diffusion space (Gong, et al., 2009a; Li, et al., 2009). Firstly, T1-weighted image of each subject was co-registered to the origin of B_0_ image, Secondly, the co-registered T1 image was normalized into MNI space. Then, the normalization parameter was inversed and applied to the AAL template in MNI space. The native template of each subject was done. This procedure has been applied in previous studies (Gong, et al., 2009a; Gong, et al., 2009b; Li, et al., 2009; Lo, et al., 2010; Shu, et al., 2009; Wen, et al., 2011). In native diffusion space, **two** nodes *i* and *j* were considered to be connected by an edge *e* = (*i, j*) if there was at least one fiber *f* between the two nodes. For each edge *e*, we calculated the connection density between the end nodes as its weight *w(e)* (Hagmann, et al., 2008):

$$w\left( e \right)=\frac{2}{S_{i}+S_{j}}\sum_{f\epsilon F_{e}} \frac{1}{l\left( f \right)}$$

where *S_i_* and *S_j_* denote the area of surfaces of AAL regions *i* and *j*, that interested with the white matter respectively, *F_e_* denotes the set of all fibers connecting regions *i* and *j* and hence contributing to the edge *e*, and *l* (*f*) denotes the length of fiber *f* along its trajectory. Thus, we obtained a weighted anatomical network for each participant.

**References**

Cui, Z., Zhong, S., Xu, P., He, Y., Gong, G. (2013) PANDA: a pipeline toolbox for analyzing brain diffusion images. Front. Hum. Neurosci., 7:42.

Fox, M.D., Snyder, A.Z., Vincent, J.L., Corbetta, M., Van Essen, D.C., Raichle, M.E. (2005) The human brain is intrinsically organized into dynamic, anticorrelated functional networks. Proc. Natl. Acad. Sci. U. S. A., 102:9673-8.

Fox, M.D., Zhang, D., Snyder, A.Z., Raichle, M.E. (2009) The global signal and observed anticorrelated resting state brain networks. J. Neurophysiol., 101:3270-83.

Gong, G., He, Y., Concha, L., Lebel, C., Gross, D.W., Evans, A.C., Beaulieu, C. (2009a) Mapping anatomical connectivity patterns of human cerebral cortex using in vivo diffusion tensor imaging tractography. Cereb. Cortex, 19:524-36.

Gong, G., Rosa-Neto, P., Carbonell, F., Chen, Z.J., He, Y., Evans, A.C. (2009b) Age- and gender-related differences in the cortical anatomical network. J. Neurosci., 29:15684-93.

Hagmann, P., Cammoun, L., Gigandet, X., Meuli, R., Honey, C.J., Wedeen, V.J., Sporns, O. (2008) Mapping the structural core of human cerebral cortex. PLoS Biol., 6:e159.

Li, Y., Liu, Y., Li, J., Qin, W., Li, K., Yu, C., Jiang, T. (2009) Brain anatomical network and intelligence. PLoS Comput. Biol., 5:e1000395.

Lo, C.Y., Wang, P.N., Chou, K.H., Wang, J., He, Y., Lin, C.P. (2010) Diffusion tensor tractography reveals abnormal topological organization in structural cortical networks in Alzheimer's disease. J. Neurosci., 30:16876-85.

Liao, W., Zhang, Z., Pan, Z., Mantini, D., Ding, J., Duan, X., Luo, C., Wang, Z., Tan, Q., Lu, G., Chen, H. (2011) Default mode network abnormalities in mesial temporal lobe epilepsy: a study combining fMRI and DTI. Hum. Brain Mapp., 32:883-95.

Mori, S., Crain, B.J., Chacko, V.P., van Zijl, P.C. (1999) Three-dimensional tracking of axonal projections in the brain by magnetic resonance imaging. Ann. Neurol., 45:265-9.

Shu, N., Liu, Y., Li, J., Li, Y., Yu, C., Jiang, T. (2009) Altered anatomical network in early blindness revealed by diffusion tensor tractography. PLoS One, 4:e7228.

Tzourio-Mazoyer, N., Landeau, B., Papathanassiou, D., Crivello, F., Etard, O., Delcroix, N., Mazoyer, B., Joliot, M. (2002) Automated anatomical labeling of activations in SPM using a macroscopic anatomical parcellation of the MNI MRI single-subject brain. Neuroimage, 15:273-89.

Wen, W., Zhu, W., He, Y., Kochan, N.A., Reppermund, S., Slavin, M.J., Brodaty, H., Crawford, J., Xia, A., Sachdev, P. (2011) Discrete neuroanatomical networks are associated with specific cognitive abilities in old age. J. Neurosci., 31:1204-12.

**Supplementary Table 1. Demographics, Clinical Characteristics, and Cognitive Performance of Patients and Healthy Controls**

| Protocols | Groups | Before or after kidney transplantation | | | F value | P value | |
| --- | --- | --- | --- | --- | --- | --- | --- |
|  |  | Baseline | One-month | Six-month |  |  |  |
| Gender | HC | 9F/8M | 9F/8M | 9F/8M | - | - | |
|  | Patients | 7F/14M | 7F/14M | 7F/14M | - | - | |
|  | P | 0.324^a^ | 0.324^a^ | 0.324^a^ | - | - | |
| Age (years) | HC | 28.9±7.2 | 28.9±7.2 | 29.9±7.2 | - | - | |
|  | Patients | 31.5±10.0 | 32.2±9.9 | 32.7±9.9 | - | - | |
|  | t value | -0.883 | -1.133 | -0.966 | - | - | |
|  | P value | 0.383^b^ | 0.122^b^ | 0.122^b^ | - | - | |
| NCT (sec) | HC | 25.2±5.0 | 24.4±6.2 | 23.9±5.4 | 0.478 | 0.625^c^ | |
|  | Patients | 42.0±17.8 | 39.3±14.6 | 39.4±20.7 | 0.860 | 0.431^c^ | |
|  | t value | -4.125 | -4.254 | -3.297 | F=0.209, P=0.812^d^ | | |
|  | P value | **<0.001^b*^** | **<0.001^b*^** | **0.003^b*^** |  |  |  |
| DST (score) | HC | 69.1±6.3 | 75.4±7.4 | 77.8±8.6 | 15.920 | **<0.001^c*^** | |
|  | Patients | 54.2±12.8 | 57.8±12.8 | 62.0±12.7 | 17.172 | **<0.001^c*^** | |
|  | t value | 4.658 | 5.017 | 4.390 | F=0.939, P=0.396^d^ | | |
|  | P value | **<0.001^b*^** | **<0.001^b*^** | **<0.001^b*^** |  |  |  |
| LTT (s) | HC | 30.1±11.4 | 34.0±10.9 | 30.0±8.7 | 2.628 | 0.088^c^ | |
|  | Patients | 53.4±10.0 | 53.1±11.6 | 49.0±9.9 | 1.155 | 0.325^c^ | |
|  | t value | -6.709 | -5.124 | -6.197 | F=0.728, P=0.487^d^ | | |
|  | P value | **<0.001^b*^** | **<0.001^b*^** | **<0.001^b*^** |  |  |  |
| SDT (s) | HC | 29.0±5.5 | 28.8±5.4 | 28.4±.3 | 0.203 | | 0.817^c^ |
|  | Patients | 48.5±9.0 | 49.3±8.8 | 48.1±12.9 | 0.100 | | 0.905^c^ |
|  | t value | -7.837 | -8.817 | -6.172 | F=0.053, P=0.948^d^ | | |
|  | P value | **<0.001^b*^** | **<0.001^b*^** | **<0.001^b*^** |  |  |  |
| SAS (score) | HC | 27.4±5.2 | 26.2±4.3 | 25.7±3.9 | 1.616 | | 0.214^c^ |
|  | Patients | 29.2±6.0 | 29.1±6.6 | 27.1±7.4 | 1.482 | | 0.239^c^ |
|  | t value | -1.017 | -1.519 | -0.768 | F=0.333, P=0.718^d^ | | |
|  | P value | 0.316^b^ | 0.138^b^ | 0.448^b^ |  |  |  |
| SDS (score) | HC | 26.6±4.9 | 27.4±5.5 | 27.0±5.6 | 1.018 | | 0.385^c^ |
|  | Patients | 34.4±11.6 | 27.1±6.3 | 27.8±8.0 | 6.637 | | **0.007^c*^** |
|  | t value | -2.799 | 0.138 | 0.746 | F=5.724, **P=0.007^d*^** | | |
|  | P value | **0.009^b*^** | 0.891^b^ | 0.737^b^ |  |  |  |
| BUN (mg/dl) | HC | 13.4±3.0 | 12.5±2.6 | 13.3±1.8 | 1.279 | | 0.292^c^ |
|  | Patients | 67.1±22.6 | 20.6±6.9 | 18.9±5.6 | 48.469 | | **0.000^c^** |
|  | t value | -10.771 | -4.958 | -4.339 | F=40.810, **P=0.000^d^** | | |
|  | P value | **<0.001^b*^** | **<0.001^b*^** | **<0.001^b*^** |  |  |  |
| Scr (mg/dl) | HC | 0.7±0.2 | 0.7±0.2 | 0.7±0.2 | 0.285 | | 0.754^c^ |
|  | Patients | 11.6±2.8 | 1.2±0.5 | 1.2±0.3 | 136.190 | | <0.001^c^ |
|  | t value | -17.481 | -3.884 | -5.134 | F=111.797, P<0.001^d^ | | |
|  | P value | **<0.001^b*^** | **<0.001^b*^** | **<0.001^b*^** |  |  |  |
| Uric acid (umol/L) | HC | 345.9±88.0 | 346.4±110.2 | 306.7±82.8 | 6.207 | | **0.005^c*^** |
|  | Patients | 410.3±141.8 | 310.1±83.2 | 369.8±96.0 | 6.255 | | 0.008^c*^ |
|  | t value | -1.634 | 1.158 | -2.140 | F=7.929, P=0.001^d*^ | | |
|  | P value | 0.111^b^ | 0.255^b^ | **0.039^b*^** |  |  |  |
| RBC count (x10^12^) | HC | 4.7±0.5 | 4.7±0.6 | 5.1±0.6 | 7.565 | | **0.005^c^** |
|  | Patients | 3.6±0.7 | 4.1±0.4 | 4.6±0.7 | 25.940 | | **0.000^c*^** |
|  | t value | 5.809 | 3.696 | 2.233 | F=8.200, P=0.001^d*^ | | |
|  | P value | **<0.001^b*^** | **0.001^b*^** | **0.032^b*^** |  |  |  |
| Hemoglobin (g/L) | HC | 141.5±16.7 | 139.2±18.0 | 151.7±19.3 | 9.857 | | 0.000^c^ |
|  | Patients | 108.3±22.0 | 125.6±14.2 | 138.1±18.4 | 21.089 | | 0.000^c^ |
|  | t value | 5.130 | 2.620 | 2.208 | F=7.617, P=0.001^d^ | | |
|  | P value | <0.001^b*^ | 0.013^b*^ | 0.034^b*^ |  |  |  |
| Hematocrit (L/L) | HC | 0.4±0.0 | 0.4±0.0 | 0.4±0.1 | 6.942 | | 0.007^c*^ |
|  | Patients | 0.3±0.1 | 0.4±0.0 | 0.4±0.1 | 20.248 | | 0.000^c*^ |
|  | t value | 4.099 | 1.410 | 1.044 | F=7.1000, P=0.002^d*^ | | |
|  | P value | <0.001^b*^ | 0.167^b^ | 0.304^b^ |  |  |  |

Continuous data are expressed as mean and standard deviation.

Pre-KT=**pre**-kidney transplantation; KT-1m = **1-**month after kidney transplantation; KT-6m = **6-**month after kidney transplantation; DST=digital symbol test; NCT-A=number connection test-A; LTT=line-tracing test; SDT=serial-dotting test; SAS=Zung Self-Rating Anxiety Scale; SDS=Zung Self-Rating Depression Scale; HC=healthy control. BUN = blood urea nitrogen, RBC = red blood cells. Less time in NCT-A, LTT, SDT and lower scores on SAS and SDS reflect better performance, while higher DST scores indicate better performance.

a, The P values are obtained by using the x^2^ test.

b, The P values are obtained by using a two-sample t test; data in parentheses are 95% confidence interval.

c, The P values are obtained by using a one way repeated measures ANOVA test.

d, The P values are obtained by using a two-way repeated measures ANOVA

^*^ Indicates a statistically significant difference.

Statistical threshold is set at P<0.05.

**Supplementary Table 2. Demographics, Clinical Characteristics, and Cognitive Performance of Patients and Healthy Controls**

| Protocols | Subgroups | Protocols | |
| --- | --- | --- | --- |
|  |  | gender | age, years |
| HCB | HCB1 | 4F/4M | 28.9±6.8 |
|  | HCB2 | 5F/4M | 29±7.9 |
| HC1m | HC1m1 | 4F/4M | 28.9±6.8 |
|  | HC1m2 | 5F/4M | 29±7.9 |
| HC6m | HC6m1 | 4F/4M | 29.9±6.8 |
|  | HC6m2 | 5F/4M | 30.0±7.9 |
| Pre-KT | Pre-KT1 | 4F/7M | 31.3±10.9 |
|  | Pre-KT2 | 3F/7M | 31.7±9.3 |
| KT-1m | KT-1m1 | 4F/7M | 32.0±10.6 |
|  | KT-1m2 | 3F/7M | 32.4±9.6 |
| KT-6m | KT-6m1 | 4F/7M | 32.5±10.7 |
|  | KT-6m2 | 3F/7M | 32.9±9.5 |

Continuous data are expressed as mean and standard deviation.

HCB = healthy control at baseline; HC-1m = healthy control at one**-**month follow-up; HC-6m = healthy control at **six-**month follow-up; Pre-KT=**pre**-kidney transplantation; KT-1m = **1-**month after kidney transplantation; KT-6m = **6-**month after kidney transplantation.
